# Machine Learning for LTE Energy Detection Performance Improvement

**DOI:** 10.3390/s19194348

**Published:** 2019-10-08

**Authors:** Małgorzata Wasilewska, Hanna Bogucka

**Affiliations:** Department of Wireless Communications, Poznan University of Technology, 61-131 Poznan, Poland; hanna.bogucka@put.poznan.pl

**Keywords:** spectrum sensing, cognitive radio, machine learning, energy detection, k-nearest neighbors, random forest

## Abstract

The growing number of radio communication devices and limited spectrum resources are drivers for the development of new techniques of dynamic spectrum access and spectrum sharing. In order to make use of the spectrum opportunistically, the concept of cognitive radio was proposed, where intelligent decisions on transmission opportunities are based on spectrum sensing. In this paper, two Machine Learning (ML) algorithms, namely k-Nearest Neighbours and Random Forest, have been proposed to increase spectrum sensing performance. These algorithms have been applied to Energy Detection (ED) and Energy Vector-based data (EV) to detect the presence of a Fourth Generation (4G) Long-Term Evolution (LTE) signal for the purpose of utilizing the available resource blocks by a 5G new radio system. The algorithms capitalize on time, frequency and spatial dependencies in daily communication traffic. Research results show that the ML methods used can significantly improve the spectrum sensing performance if the input training data set is carefully chosen. The input data sets with ED decisions and energy values have been examined, and advantages and disadvantages of their real-life application have been analyzed.

## 1. Introduction

According to [1], the total number of world mobile subscriptions in the second quarter of 2019 was around 7.9 billion. Moreover, the predictions therein show that, in 2024, the global mobile data traffic will amount to approximately 130 Exabytes per month, and 5G networks will carry 35 percent of that traffic. There will be 22.3 billion connected devices constituting the Internet of Things, out of which 4.1 billion will be connected via cellular networks. These facts and predictions imply challenges for planning radio resource allocation for wireless connections, and require effective, possibly cognitive methods of spectrum access.

The concept of Cognitive Radio (CR) includes radio devices capable of learning and adapting to external conditions. The aim is to increase the use of radio resources in consideration of the constantly growing number of wireless transmitters. CR users are called Secondary Users (SUs), while licensed systems (in a given frequency band) users are called Primary Users (PUs). SUs try to achieve radio environment awareness in order to opportunistically access the radio resources, temporarily not used by PUs. Radio-environment awareness, in particular, the awareness of spectrum occupancy and transmission conditions, enable the optimization of the SUs’ signal transmission. This allows for the maximization of spectrum usage, while keeping the interference level observed by PUs in the acceptable range. Spectrum Sensing (SS) commonly refers to the multiplicity of methods of obtaining the spectrum-occupancy awareness of SUs. Based on this awareness, CR should make intelligent decisions on transmission—and reception—related actions, and constantly improve these decisions by learning from past experience. For this purpose, Machine Learning (ML) methods can be used.

There are some conventional SS methods, such as Energy Detection, Matched Filtering, and Cyclostationarity Detection [2]. Energy Detection (ED) is the simplest method, in which the received signal power is compared with a specific threshold to determine the presence of a (noisy) PU’s signal or just the noise. In contrast to Cyclostationarity Detection [3], Matched Filtering [4], and most other methods, it does not require any specific knowledge on the signal that is to be detected; however, it does require the knowledge of the channel noise power. ED methods are considered in [5,6,7,8]. Kim et al. [5] propose a histogram-based method to determine the threshold of energy detection. Digham et al.’s study [6] concerns ED over fading channels, and the signal of unknown characteristics is also under consideration, the same as in [7] and [8]. The mentioned articles discuss the ED method with a single decision threshold. A double-threshold ED has been discussed in [9,10,11]. Although, in many papers, the noise power is assumed to be known, some works present the ways of noise estimation for ED purposes. Farag et al. [12] propose dynamic threshold evaluation based on noise estimation. Furthermore, papers [13,14,15] present other methods of noise-power estimation for energy detection.

ML techniques have also been proposed as methods of SS performance improvement. ML methods can be used directly as detection algorithms. Subekti et al. [16] propose a Deep Autoencoder Neural Network and Support Vector Machine (SVM) algorithms as a way of learning signal features and then classifying signals as PU signal or SU signal. The SVM and different Neural Network algorithms are also presented in [17]. ML can be also used together with one of the SS methods, for example with ED. ED-based ML for Spectrum Sensing is presented in [18], which employs an SVM-based classifier for SS enhancement. ED and ML is also a popular combination used in cooperative sensing [19,20,21,22]. Tavares et al. [19] use three Bayesian SS estimators and compares them with typical cooperative SS methods, such as Maximum Ratio Combining, AND and OR rules. Ma et al. [20] propose using the Extreme Learning Machine (ELM) for channel pattern classification in cognitive radio networks with multiple PUs. The proposed method uses ELM in the fusion center to correctly classify the channel state. Mustafa et al. [21] propose using a Neural Network as a decision fusion scheme in the fusion center. Mikaeil et al. [22] analyze the k-Nearest Neighbors (kNN), Decision Tree (DT), SVM and Naive Bayes (NB) ML algorithms as decision-making methods in the fusion center. ML using calculated signal energy values in the form of energy vectors is presented in [23,24,25]. Algorithms K-means clustering and SVM are used in [23] for discovering PU’s transmission patterns and statistics and for SS, respectively. Variational Bayesian learning for the Gaussian mixture model is used in [24] as an SS method for a multi-antenna CR network. In both of those papers, noncooperative SS is considered. Cooperative SS is considered in [25], where SVM, kNN and NB algorithms are used for signal classification.

The goal of this work is to show how much the probability of detection can be increased by using ML methods as a supporting component in autonomous (noncooperative) spectrum sensing. Below, SS is used to detect occupied Long-Term Evolution (LTE) downlink signal Resource Blocks (LTE-RBs) utilized by a base station (eNodeB). The ultimate goal is to create a possibility of transmitting the CR signal in the Fifth Generation (5G) New Radio (NR) standard with a flexible choice of RBs orthogonal to LTE-RBs.

Thus, here, ED is used as the first stage of LTE downlink signal-presence detection in a given area. In the considered network, the communication traffic is characterized by a certain daily distribution of intensity. Moreover, due to LTE frequency planning and channel characteristics in the considered geographical area, some of the frequency resources are used with location-dependent probability. Shadowing characteristics also reflect spatial correlation. There are patterns and dependencies of LTE-RBs utilization in the time-, frequency- and spatial domains. Hence, in the second stage of SS, ML is used to discover these dependencies and increase detection probability while decreasing the probability of false alarm. Two methods of ML are considered: kNN and Random Forest (RF). Both algorithms are studied and compared in terms of the SS performance improvement. As for the ML input data, both the ED output decisions data, and the detected spectrum energy values are considered, and the advantages and disadvantages of using them in a real-world scenario are discussed.

The rest of the paper is organized as follows. In Section 2, the basics of ED are described. Section 3 presents the considered ML methods and their application in our SS scenario. In Section 4, our novel algorithm for improved LTE-RBs detection based on ED and ML is explained. Section 5 presents the simulation system with the applied methods, and discusses the experimental results. Finally, the key findings and conclusions are summarized in Section 6.

## 2. Spectrum Occupancy Autonomous Detection

Spectrum occupancy detection is based on choosing between two possible hypotheses. Hypothesis $H_{0}$ assumes that the received signal consists of noise only, whilst hypothesis $H_{1}$ means that the received signal is a sum of noise and the PU’s transmitted signal distorted by the channel, i.e.,

(1) $$\begin{array}{cc} H_{0}:y\left(t\right) & =n(t),\\ H_{1}:y\left(t\right) & =h(t)*s(t)+n(t), \end{array}$$

where y(t) is a received signal, s(t) is the transmitted signal, h(t) is the channel impulse response, n(t) is the noise, and * denotes linear convolution. The goal of sensing is to find out which hypothesis is more probable. The right sensing algorithm should maximize the probability of correct detection ($P_{d}$), while maintaining the value of the probability of false alarm ($P_{\mathrm{fa}}$) close to some assumed level. Probability $P_{d}$ is defined as the probability of (correctly) deciding through spectrum sensing that hypothesis $H_{1}$ is true. Probability $P_{\mathrm{fa}}$ is the probability of making a wrong decision that hypothesis $H_{1}$ is true, while in fact hypothesis $H_{0}$ is true.

Energy detection is one of the popular SS methods, relatively not computationally complex. However, it requires the knowledge of the noise power level, and it works poorly in low SNR regions. In the ED method, the energy of the received signal is calculated in some given time and frequency range. The calculated energy of signal y(n) is a so-called test function T(y). For *N* samples of the received complex signal y(n), the test function is defined as:

(2) $$T\left(y\right)=\frac{1}{N}\sum_{n=1}^{N}{\left|y\left(n\right)\right|}^{2}.$$

In order to decide whether the spectrum is occupied or not, the test function value is compared with the threshold, given by equation:

(3) $$\lambda =\sigma_{n}^{2}\left(Q^{-}1\left({\bar{P}}_{\mathrm{fa}}\right)\sqrt{\frac{1}{N}}+1\right),$$

where $\sigma_{n}^{2}$ is the noise power, ${\bar{P}}_{\mathrm{fa}}$ is the assumed level of the probability of false alarm, and $Q^{-}1\left(\cdot \right)$ is the inverse Q function, which is given by:

(4) $$Q\left(x\right)=\frac{1}{\sqrt{2\pi }}\int_{x}^{\infty }\left(-\frac{u^{2}}{2}\right)du,$$

where variable *u* is a variable of integration. If the value of T(y) is higher than threshold λ, the decision that the spectrum is occupied is made. Otherwise, the spectrum is considered unoccupied (”free”). Thus, the probabilities of detection $P_{d}$ and $P_{\mathrm{fa}}$ are defined as:

(5) $$\begin{array}{cc} \; & P_{d}=\mathrm{Pr}\left\{T\left(y\right)>\lambda |H_{1}\right\},\\ \; & P_{\mathrm{fa}}=\mathrm{Pr}\left\{T\left(y\right)>\lambda |H_{0}\right\}. \end{array}$$

To summarize, the result of the first stage of our algorithm is the binary decision on whether $H_{0}$ or $H_{1}$ is true. However, we will also consider the use of T(y) for the second stage.

## 3. Machine Learning for Improved Spectrum Sensing

Typically, ML methods are used to achieve a near-optimum solution of the optimization problem, when it is too complex for the conventional optimality analysis and when the function mapping the input data to the output (the solution of the problem) is unknown. In the radio communication systems, this kind of uncertainty is caused by the noise (thermal, colored, impulse or other) or other types of channel distortions and interference. In this paper, ML is applied as a way of fine sensing decision enhancement. Based on the specific LTE-RBs -occupancy decision from the first stage (ED decision, T(y) value, range of the LTE subcarriers, time slot, adjacent LTE-RBs occupancy etc.). The ML algorithm should be able to deduce whether currently examined LTE-RBs are busy or idle. Therefore, we shall use the *classification* algorithms that classify the input data for the final binary decision. Let us consider two ML classification algorithms: the k-Nearest Neighbors and Random Forest. The reasoning behind selection of these two is provided in the subsections below.

### 3.1. k-Nearest Neighbors

**Figure 1:**
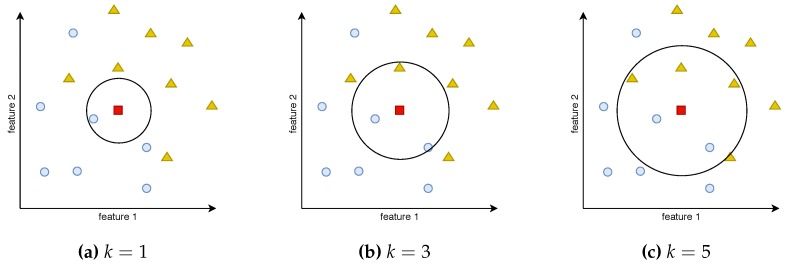
k-Nearest Neighbors—visualization of the closest data points for different *k* values.

KNN based classification is considered as one of the simplest ML algorithms [26]. It is a supervised algorithm, which means that, in the training phase, it requires full knowledge of the output data corresponding to the training input data. Based on the training data set, new input data can be classified into one of the output categories by calculating the distances (usually the Euclidean distance) to *k* closest neighbors in the used features space [27]. For example, in the case of parameter k=1, only one closest data point is considered, and its output value is assigned to the label of the data point that is supposed to be classified. In the case of k>1, the most numerous group of the neighboring points of one category determines the result. Figure 1 shows the example training data points of two categories: blue circles and yellow triangles arranged in the space of two features. When a new point (red square) from outside of the training set occurs, and an output value is to be determined, the algorithms looks for *k* closest neighboring data points. Figure 1a shows that the closest neighbor of the red square is a blue circle, so, for k=1, the red square is classified as a blue circle. In addition, for k=3 (Figure 1b), two out of three closest points belong to the blue circle category. However, for k=5 shown in Figure 1c, there are three yellow triangles and only two circles closest to the input data point, so the square is classified as a yellow triangle.

In typical LTE downlink transmission (as in our considered scenario), LTE-RBs are being assigned to users in groups of several adjacent LTE-RBs, depending on a user’s demand associated with a specific service. This is because the LTE scheduler tries to avoid fragmentation of resources for a user. This means that the probability that a given LTE-RB is occupied is higher if the adjacent LTE-RBs (in the time domain or in the frequency domain) are also occupied. The occupation of the tangential LTE-RBs is not as probable. Therefore, the classification of LTE-RBs based on closest neighbors, namely kNN, is an appropriate method that should provide some detection improvement. However, *neighborhood* and distance, in this case, should be carefully re-defined. These issues are considered in the following section. Finally, note that the kNN algorithm requires storing all the training data, and the prediction can be very slow for large sizes of training datasets and high *k* parameter values.

### 3.2. Random Forest

**Figure 2:**
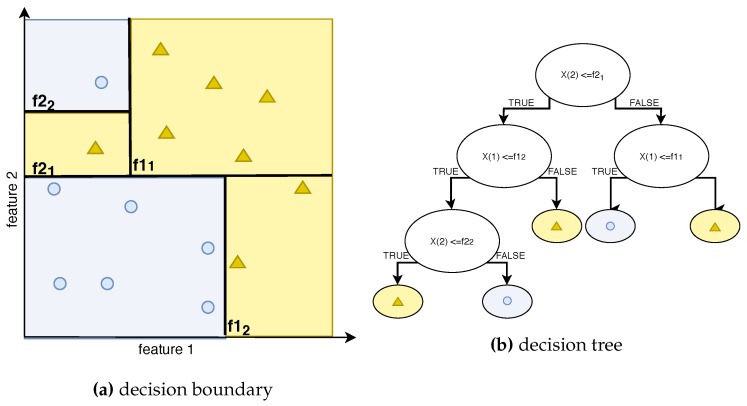
Decision tree—tree with depth 3.

Random Forest in an expanded version of the DT algorithm [28]. In this work, the classifying version of RF is used. RF consists of a number of single DTs. The DT algorithm divides the input data set into further subsets, each of which means a different output category (for example, blue circles or yellow triangles, as in our example above). A DT algorithm of full depth iteratively divides a feature space into subspaces, as shown in Figure 2a, so that every training data point belongs to a subspace of its own category. Figure 2b presents a decision-making algorithm for a new input data point of two features X=[X(1),X(2)]. Thanks to feature subspaces’ boundaries specified by the DT algorithm (values $f1_{1},f1_{2},f2_{1},f2_{2}$), a new data point can be assigned to an output label of a blue circle or a yellow triangle.

The main drawbacks of DTs are that the algorithm gets very complex for big data-sets, and has a tendency to overfit. The tree that assigns all training data points correctly most likely is of large depth and highly overfits, i.e., results in boundaries that are too sharp for different data sets. One way of preventing this is to use trees of smaller depths that may not work with 100% accuracy on the training data but perform better on new input data and are also less complex. Another way is to use the RF algorithm, which creates a number of DTs with slightly different boundaries. Single DTs used in RF might overfit on the training data, but each of them does it in a different way, which creates an averaged result after combining separate trees and prevents overfitting [29].

In our considered scenario, the occupied and unoccupied LTE-RBs are likely to create regions on the time-frequency plane (also in the location domain). Therefore, the RF algorithm is proposed as an alternative for the kNN algorithm. RF does not require storing as much data as kNN, so it works faster but is still easy to implement and analyze. Algorithms as kNN and RF seem to be appropriate for the kind of the considered problem, where there are simple dependencies between data, but still too complex to analyze with standard optimization and other, even heuristic, non-ML methods.

## 4. New Algorithm for Improved LTE-RBs Energy Detection

Based on the considerations on the applicability of kNN and RF methods for improved spectrum sensing presented above, let us now propose the LTE-downlink signal-presence detection algorithm. The first step of the algorithm is either the calculation of the energies of subsequent blocks of *N* received signal samples (forming a vector of energy values for a given data set) in a given frequency range, or hard decisions on signal presence based on these energy values (forming a vector of binary values). Then, one of the considered ML algorithms is applied, i.e., either kNN or RF. For the proper implementation of this ML algorithm stage, features calculation is necessary. Every feature is calculated for every resource block in every time slot equal to the duration of a single LTE-RB. The following features are used in our proposal:

1. the index of a time slot (the smallest LTE-RB dimension in the time domain),
2. the index of the LTE basic subcarriers set (the smallest LTE-RB dimension in the frequency domain; in LTE, consisting of 12 OFDM (Orthogonal Frequency Division Multiplexing) subcarriers),
3. ED hard decision—values 0 or 1, or, alternatively, the energy value—a real number,
4. the number of diagonal (tangential) neighboring LTE-RBs detected as busy or alternatively, the sum of energies of diagonal (tangential) neighboring LTE-RBs,
5. the number of adjacent neighboring LTE-RBs detected as busy or alternatively, the sum of energies of adjacent neighboring LTE-RBs.
6. history coefficient with forgetting factor

The use of listed features is intended for improving the ML algorithm performance by feeding it enough information about LTE-RB occupation that ML prediction applies to, and about the state of the closest neighboring LTE-RBs.

When ED hard decisions are used in the algorithm, the history coefficient with the forgetting factor is calculated as follows:

(6) $$\phi_{ED}\left(m,l\right)=ED\left(m,l\right)+\alpha \cdot \phi_{ED}\left(m-1,l\right),$$

where ED(m,l) is the energy detection decision for LTE-RB for which *m* is a time slot index, *l* is the index of the LTE subcarriers set, and α is the forgetting factor in the range of $\left[0,\ldots ,1\right]$.

In the case of using EV as inputs to the ML stage, the history coefficient is calculated as:

(7) $$\phi_{E}\left(m,l\right)=E\left(m,l\right)+\alpha \cdot \phi_{E}\left(m-1,l\right),$$

where E(m,l) is the energy value for the LTE-RB of time slot *m*, and frequency index *l*.

**Figure 3:**
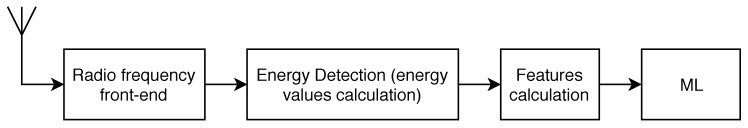
System model.

To illustrate data-flow in the algorithm, Figure 3 is presented that shows the system model on the receiver side.

**Figure 4:**
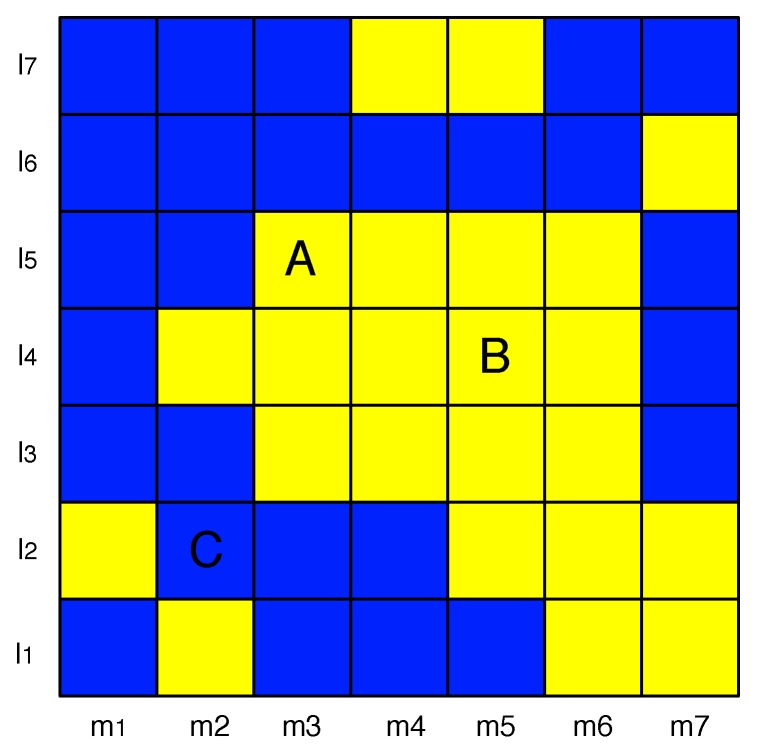
LTE Resource Blocks features.

To illustrate the features calculation, Figure 4 is presented. It shows LTE-RBs in the time- and frequency domains as yellow and blue squares, which represent resources detected as occupied and free, respectively. For example, for the LTE-RB marked as **A**, the index of the time feature is $m_{3}$, and the frequency index is $l_{5}$. The ED algorithm decided that LTE-RB **A** is occupied, so the third feature can be represented as number 1. There is also one tangential neighboring LTE-RB that is marked as occupied by ED, and one adjacent occupied LTE-RB. Assuming that $m_{1}$ is the first time slot index, the history coefficient for **A** is equal to 0. Thus, the feature set for a LTE-RB **A** can be presented as a feature vector: $\left[m_{3},l_{5},1,1,1,0\right]$. Similarly for LTE-RB marked as **B**, a feature vector can be presented: $\left[m_{5},l_{4},1,2,2,\phi_{ED}\left(m_{5},l_{4}\right)\right]$, and for LTE-RB **C**: $\left[m_{2},l_{2},0,0,1,\phi_{ED}\left(m_{2},l_{2}\right)\right]$.

Similar features can be calculated engaging energy values instead of ED decisions. Then, most of the features will not be represented as discrete numbers anymore, but as real numbers.

## 5. Simulation Experiment

### 5.1. Simulation Setup

In order to test our ML improved LTE-RB energy detection algorithm, a downlink LTE signal (OFDM signal) has been generated for the LTE bandwidth of 10 MHz. This bandwidth is divided into 50 LTE-RBs. Each LTE-RB has the standard bandwidth of 180 kHz and is transmitted over 12 OFDM subcarriers. The LTE-RB time-slot lasts 0.5 ms. In order to generate an OFDM symbol, the order 1024 Inverse Fast Fourier Transform (IFFT) algorithm is used over random BPSK-modulated data symbols. There are seven OFDM symbols per each LTE-RB. The OFDM cyclic prefix has the length of 144 samples.

**Figure 5:**
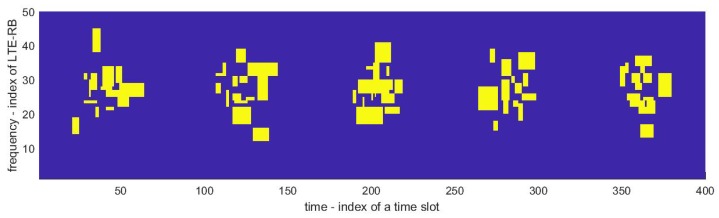
Transmitted LTE Resource Blocks.

One of the reasons for using ML in spectrum sensing in the proposed solution is to discover and make use of the time intervals when the communication traffic is low, and minimize the probability of false alarm in these intervals that would enable SUs to transmit. To reflect communication traffic variations, some random periodicity has been introduced in the generated signal. It has been assumed that, for some periodically occurring time slots, the probability of transmission is higher than for others. For these time intervals, it is assumed that LTE-RB occupation follows the normal distribution. Thus, for time slots of lowest communication traffic, the probability of transmitting by PU is much lower. Moreover, in the frequency domain, some regularities have also been introduced. They reflect typical LTE frequency planning, where, in a given area, some of the frequency bands are less likely to be used due to specific (adverse) channel conditions and Inter-Cell Interference Coordination (ICIC) schemes. Here, for the simplicity of implementation, it has been assumed that the probability of LTE-RBs occupation is higher in the central frequency range of the considered bandwidth. It is justified by assuming that the RB scheduler reduces interference from the neighboring frequency ranges. Figure 5 presents an example of the generated busy and idle LTE-RBs in the time and frequency domains. The yellow color represents occupied LTE-RBs and blue represents idle ones.

The generated transmission signal is sent over a multi-path Rayleigh fading radio channel with the shadowing effect and Additive White Gaussian Noise (AWGN). The extended pedestrian A Model (EPA) [30] has been chosen as a multi-path channel model. The shadowing effect has been simulated using log-normal distribution in the considered area. The signal-to-noise ratio (SNR) is calculated as a mean value of the signal power in one time slot to noise power observed in this time slot.

The received LTE-downlink signal is then the subject of our LTE spectrum sensing Method described in Section 4. Four basic versions with various parameters have been examined: combinations of using EV or ED hard decisions as the ML input data set and using kNN or RF as the actual algorithm for improving detection. The kNN has been tested for the parameter *k* values equal to 1, 3, 5 and 9. Random Forest has been tested for one tree, 10, 50 and 100 trees. Those parameter values were chosen heuristically as it was observed that, for higher parameter values, the results were getting worse. The number of samples used in training and testing was equal to 328,000, which corresponds to LTE-RBs transmitted in 6560 time slots. For each of those 328,000 LTE-RBs, a set of features has been generated. During testing, it was important to maintain groups of samples together, as the data is correlated and in time in frequency. For validation purposes, the group k-fold cross-validation was performed. Training sets were composed of 292,000 samples, while testing sets were composed of 36,000 samples. Finally, let us report that based on the conducted trials, the forgetting factor α equal to 0.9 has been selected.

For the simulation of the LTE signal, channel, ED stage and all of the features calculation, Matlab software (R2018a version 9.4, MathWorks, Natick, MA, USA) has been used, along with the Communications System Toolbox and Statistics and Machine Learning Toolbox. ML algorithms have been implemented using the scikit-learn library in Python [31]. In order to facilitate reproducibility of the experiments, supplementary materials including generated datasets used in the simulations are available online, and can be found in [32]. There are two datasets containing sets of features for LTE-RBs, one containing all of the mentioned features in Section 4, along with some additional ones, and a second set of features containing additional features of localization in space, generated for a simulation with the shadowing channel. More details can be found in the feature-set description available online.

### 5.2. Simulation Results

**Figure 6:**
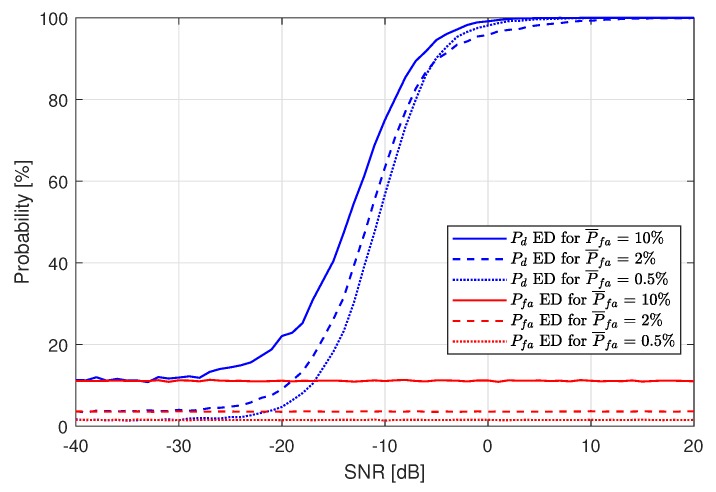
Probability of detection $P_{d}$ for the Energy Detection stage for ${\bar{P}}_{\mathrm{fa}}=10\%$, ${\bar{P}}_{\mathrm{fa}}=2\%$ and ${\bar{P}}_{\mathrm{fa}}=0.5\%$.

Let us now present and discuss the simulation experiment results. First, ED has been performed for different assumed probabilities of false alarm (denoted as ${\bar{P}}_{\mathrm{fa}}$), without the use of any ML algorithm. The results are shown in Figure 6. For each ${\bar{P}}_{\mathrm{fa}}$, one can observe that $P_{d}$ is always higher than $P_{\mathrm{fa}}$. As expected, $P_{\mathrm{fa}}$ remains constant, and approximates the ${\bar{P}}_{\mathrm{fa}}$ value for each $P_{d}$ plot for every SNR value from the examined SNR range.

**Figure 7:**
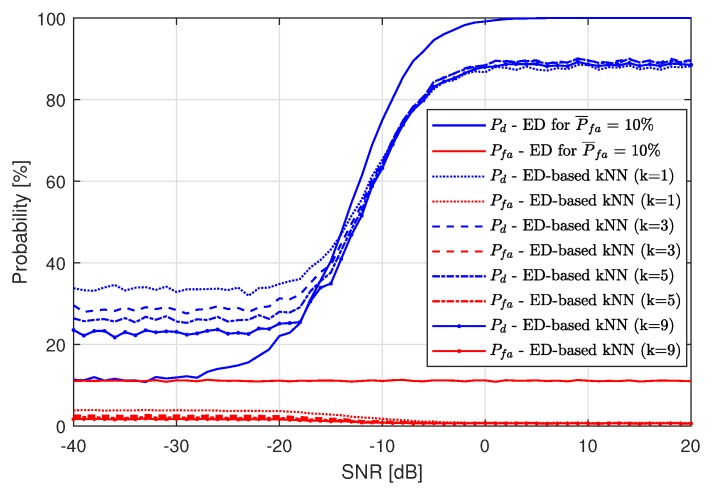
Resulting probability of detection $P_{d}$ and probability of false alarm $P_{\mathrm{fa}}$ of the Energy Detection-based k-Nearest Neighbors method for ${\bar{P}}_{\mathrm{fa}}=10\%$.

Then, two ML algorithms have been used: the kNN algorithm and the RF algorithm. Both of them were examined in two cases: ED-based and EV-based. ED-based ML means that ML features were calculated based on ED results, and for EV-based ML features were calculated using energy values. Figure 7 presents the results of the ED-based kNN algorithm for the threshold λ set only for ${\bar{P}}_{\mathrm{fa}}=10\%$. This value of ${\bar{P}}_{\mathrm{fa}}$ has been chosen since the differences between simulation results are most visible in that case. Other values of ${\bar{P}}_{\mathrm{fa}}$ are also analyzed later on in the paper. There, the probability of detection $P_{d}$ and the probability of false alarm $P_{\mathrm{fa}}$ at the output of the first (denoted as ED in the legend) and the second stage of the algorithm are presented. Note that the false alarm probability ${\bar{P}}_{\mathrm{fa}}$ assumed for the first stage of the algorithm (energy detection) may differ from the resulting value of $P_{d}$. It can be observed that the best performance in terms of $P_{d}$ has been achieved for k=1, which means that the simplest version of the kNN algorithm works best. In the case of assumed ${\bar{P}}_{\mathrm{fa}}=10\%$ in the ED algorithm, kNN improves SS performance for lower values in the considered SNR range. For SNR values higher than −15 dB, the kNN results are worse than the ED results.

**Figure 8:**
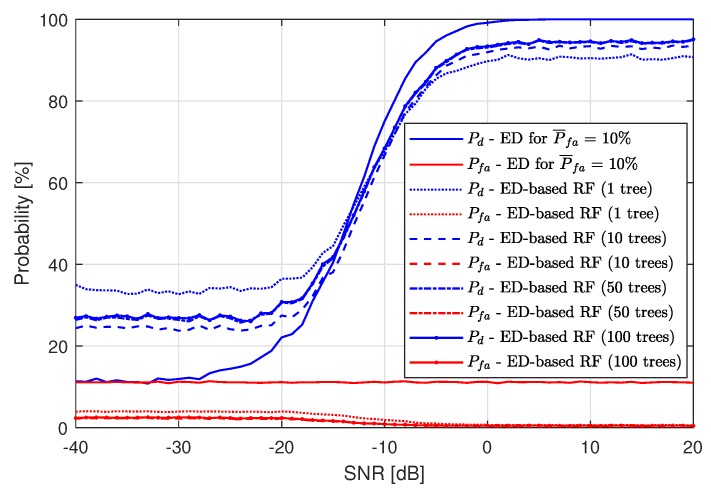
Resulting probability of detection $P_{d}$ and probability of false alarm $P_{\mathrm{fa}}$ of the Energy Detection-based Random Forest method for ${\bar{P}}_{\mathrm{fa}}=10\%$.

For the RF algorithm applied, the results are best for one tree used. Figure 8 presents the RF application (after the first ED stage) performance compared to single-stage ED for ${\bar{P}}_{\mathrm{fa}}=10\%$. As in the case of using the kNN algorithm, RF also improves the spectrum sensing probability for low SNR values, but performs worse than just ED for high SNRs. The low performance for high SNR is caused by $P_{\mathrm{fa}}$, which is the cause of machine learning algorithm fallibility. For high SNRs, all of the LTE-RBs wrongly recognized as busy by ED are gathered around actually busy LTE-RBs. Since ML is trained to recognize grouped LTE-RBs marked as busy by ED as more probable to contain a signal, decreasing $P_{\mathrm{fa}}$ is the cause of the increasing number of detection errors. The higher the value of $P_{\mathrm{fa}}$, the lower the maximum value of $P_{d}$. In Figure 7 and Figure 8, one can see that the maximum achieved value of $P_{d}$ by both ML algorithms is approximately equal to 90%, which means that the maximum $P_{d}$ approximately equals $100\%-{\bar{P}}_{\mathrm{fa}}$.

**Figure 9:**
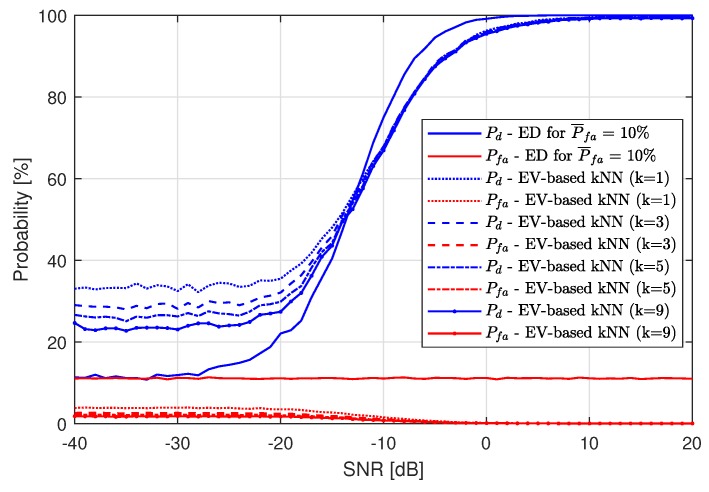
Resulting probability of detection $P_{d}$ and probability of false alarm $P_{\mathrm{fa}}$ of the Energy Vector-based k-Nearest Neighbors method for ${\bar{P}}_{\mathrm{fa}}=10\%$.

With the second feature set used in the proposed detection algorithm, energy values calculated for separate LTE-RBs, along with all of the other features calculated based on them, have been used directly in ML. Figure 9 shows the results of the kNN algorithm (again applied as the second stage of LTE-RB detection) compared to single-stage ED results with ${\bar{P}}_{\mathrm{fa}}=10\%$. It should be noted that $P_{\mathrm{fa}}$ does not affect the ML results in this case. Again, the best results have been obtained for k=1. This time, the application of ML results in achieving detection probability equal to 100% for high SNR values. In the whole SNR range, applied ML improves the performance of ED.

**Figure 10:**
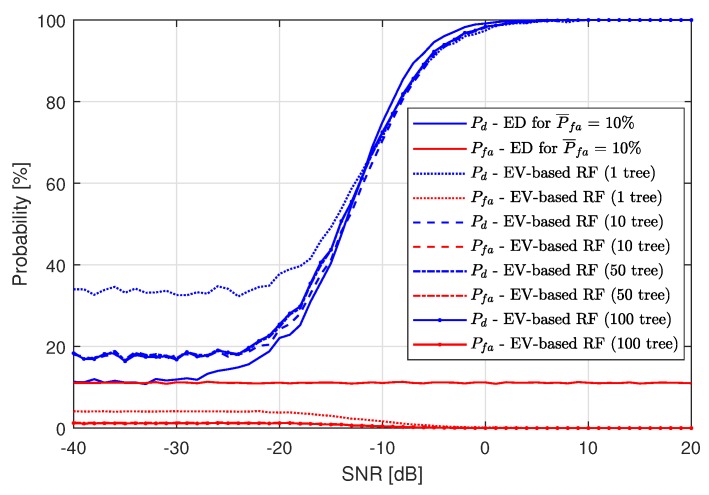
Resulting probability of detection $P_{d}$ and probability of false alarm $P_{\mathrm{fa}}$ of the Energy Vector-based Random Forest method for ${\bar{P}}_{\mathrm{fa}}=10\%$.

The same analysis has been performed for the RF algorithm. Again, one tree seems to be the best choice for our SS scenario. The results are presented in Figure 10.

**Figure 11:**
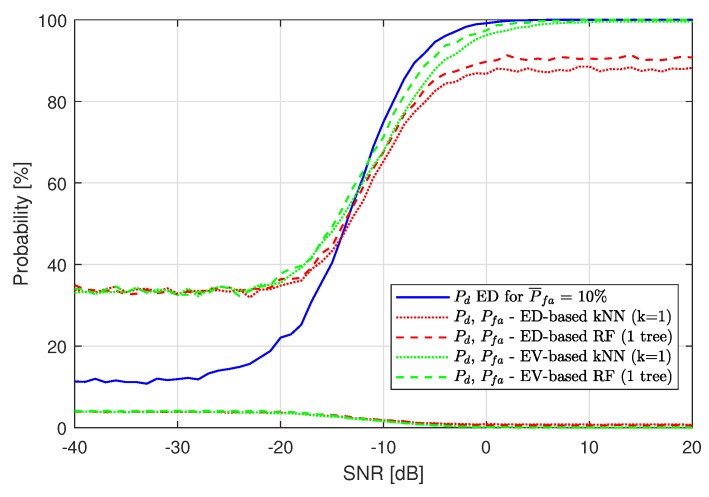
Probability of detection $P_{d}$ comparison of the Energy Detection-based and Energy Vector-based k-Nearest Neighbors and Random Forest methods for ${\bar{P}}_{\mathrm{fa}}=10\%$.

To compare the best results of the ML application to our SS scenario, Figure 11 is presented. Here, ED results for $P_{\mathrm{fa}}$ are compared with the kNN and RF algorithm used on the ED feature set (red plots) and with kNN and RF used on the EV feature set (green plots). ML algorithms improve SS approximately the same in all cases for low SNR, but, for high SNR values, the difference is significant and shows the superiority of EV-based ML over ED hard-decision-based ML.

**Figure 12:**
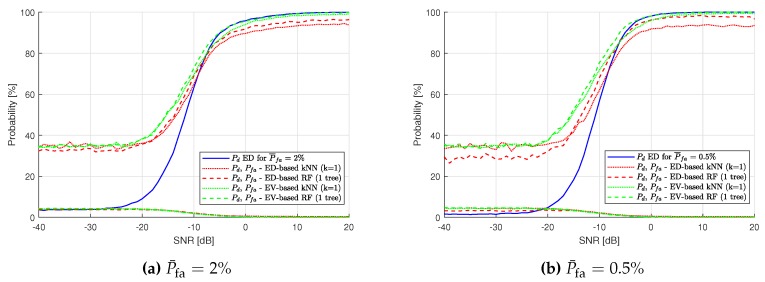
Probability of detection $P_{d}$ comparison of the Energy Detection-based and Energy Vector-based k-Nearest Neighbors and Random Forest methods for different assumed ${\bar{P}}_{\mathrm{fa}}$.

Similar figures have been obtained for lower values of ${\bar{P}}_{\mathrm{fa}}$ used in ED, namely ${\bar{P}}_{\mathrm{fa}}=2\%$ (Figure 12a) and ${\bar{P}}_{\mathrm{fa}}=0.5\%$ (Figure 12b). For smaller ${\bar{P}}_{\mathrm{fa}}$, it is visible that the results for ED-based ML and EV-based ML become similar, although EV-based ML performance remains better. For ED-based ML, the impact of ${\bar{P}}_{\mathrm{fa}}$ is still showing up. In Figure 12a, the maximum ${\bar{P}}_{d}$ value of the red plots is close to 97%, and, in Figure 12b $P_{d}$, the results are similar. The results of EV-based ML all reach 100%.

Having compared the results, one can draw the conclusion that EV-based ML methods applied in spectrum sensing, in our scenario, perform better than ED-hard-decision-based ML methods, so there is no reason to use ED hard decision data in the feature set. Although using energy directly in ML has significant advantages, it also has some disadvantages. For example, storing and processing energy values requires more memory than storing binary numbers as in ED-decision-based ML. For EV, the more accurate the energy values, the more precise the results. ED can be implemented using simple analog components with a comparator to compare the measured energy with a given voltage set as a threshold. At the output of a similar EV setup, an analog-to-digital converter is needed, which would also introduce a quantization error to the energy value.

Finally, note that, in the case of ED-based ML, the results may be poorer (in terms of the lower probability of detection) for high SNR values than for the ED algorithm applied alone. This could be solved by using the ML stage adaptively. In that case, ML could be used only for low SNR values, and, for high SNRs, the ED performance should be sufficient. This requires the implementation of some adaptation algorithm or components, but also results in reduced algorithm complexity for high SNRs. Hence, it shortens the processing time.

Table 1 compares the advantages and disadvantages of using ML based on ED data, and ML based on EV data.

**Figure 13:**
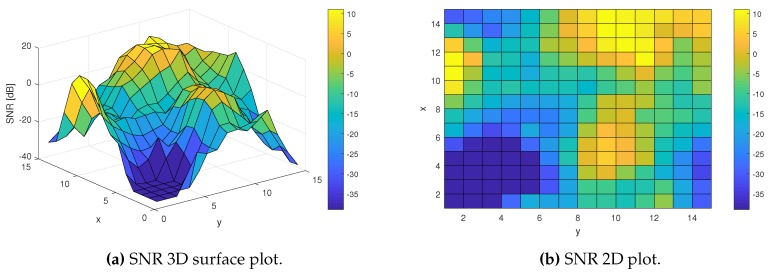
SNR values resulting from the shadowing effect in the considered area.

The proposed detection methods using ML both need to learn separately for different SNR values. This requires some knowledge on the SNR level at the specific moment. The noise level has to be estimated anyway for ML using ED hard-decisions as the input data, but ML based on EV data should not need to require noise estimation. In order to solve this problem, the ML algorithm can learn separately for different location points, assuming that the shadowing variations in the channel are much slower than the Rayleigh fading. In order to check this method, a shadowing channel model was generated. Figure 13 shows the SNR values for the generated example of a shadowing channel characteristic in different location points. It consists of values ranging from around −40 dB to 10 dB, which covers the whole range of SNR values for which $P_{d}$ ranges from its minimum value to 100%. For this simulation experiment, LTE data of the same parameters as used in the previous experiments have been transmitted and collected at every point of 15-by-15 location area. Then, the ED sensing method and ML methods have been applied separately at every one of 225 location points, in order to check if ML is able to learn correctly to improve detection at different location points.

**Figure 14:**
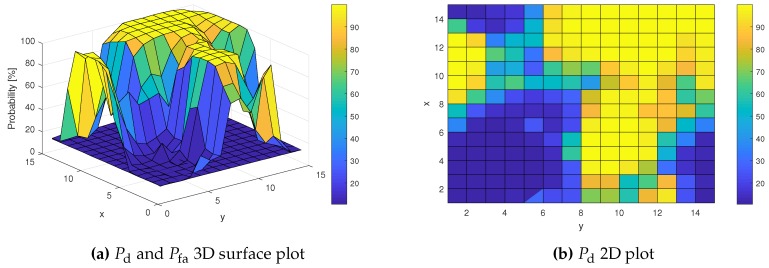
$P_{d}$ and $P_{\mathrm{fa}}$ for different locations.

Probabilities $P_{d}$ and $P_{\mathrm{fa}}$ of the ED method applied alone are presented in Figure 14. These results have been obtained for ${\bar{P}}_{\mathrm{fa}}=10\%$. As expected, $P_{d}$ values range from 10% to 100%.

**Figure 15:**
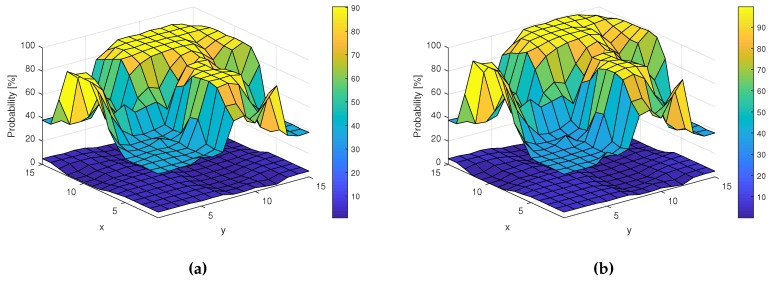
$P_{d}$ and $P_{\mathrm{fa}}$ for the k-Nearest Neighbors method applied in different locations. (**a**) $P_{d}$ and $P_{\mathrm{fa}}$ surfaces for Energy Detection-based k-Nearest Neighbors; (**b**) $P_{d}$ and $P_{\mathrm{fa}}$ surfaces for Energy Vector-based k-Nearest Neighbors.

Again, algorithms kNN and RF have been applied to compare ML results on ED data and EV data. Figure 15 shows $P_{d}$ and $P_{\mathrm{fa}}$ surfaces for ED input (Figure 15a) and EV (Figure 15b) for the kNN algorithm with parameter k=1.

**Figure 16:**
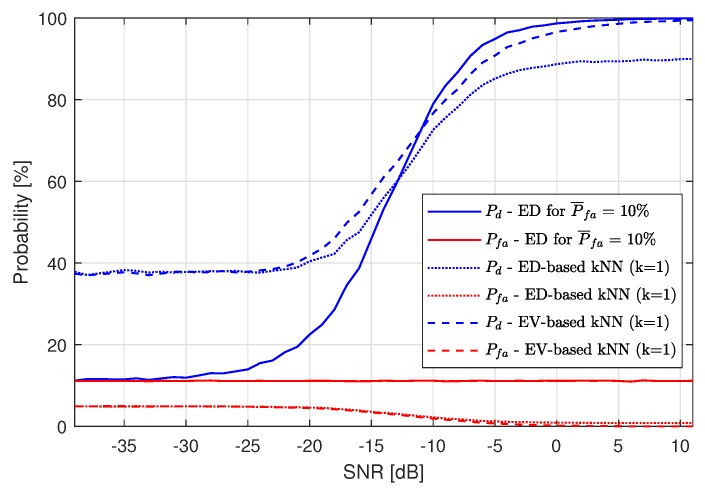
Resulting probabilities $P_{d}$ and $P_{\mathrm{fa}}$ of Energy Detection-based k-Nearest Neighbors compared with Energy Vector-based k-Nearest Neighbors for ${\bar{P}}_{\mathrm{fa}}=10\%$ for different SNR values with a shadowing channel.

The probability of detection $P_{d}$ for kNN based on ED hard-decision input ranges from 10% to 90%, while $P_{d}$ for kNN based on EV input ranges from around 37% to 100%, so the improvement resulting from using EV data is visible. This is even more clear when $P_{d}$ is plotted versus SNR in Figure 16.

**Figure 17:**
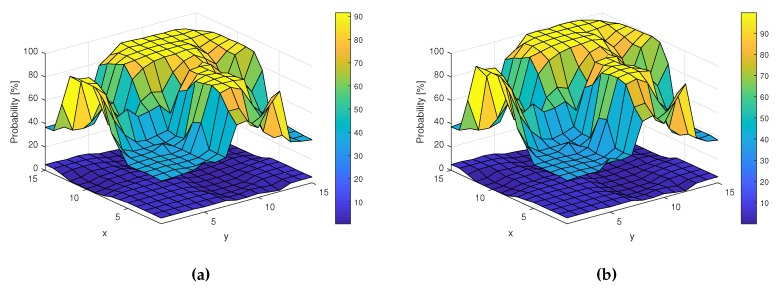
Probabilities $P_{d}$ and $P_{\mathrm{fa}}$ in different locations for the applied Random Forest method. (**a**) $P_{d}$ and $P_{\mathrm{fa}}$ surfaces for Energy Detection-based Random Forest; (**b**) $P_{d}$ and $P_{\mathrm{fa}}$ surfaces for Energy Vector-based Random Forest.

These results have been obtained by calculating the SNR values for every location point. The results are similar to those achieved without shadowing (assuming that these SNR variations can be corrected by adaptive gain control) or even slightly better, as, for low SNR values, $P_{d}$ is equal to 37%, while, for the previous experiments, $P_{d}$ is equal to around 34%. Similar results have been obtained for the RF algorithm using one decision tree. Figure 17 shows the surfaces of $P_{d}$ and $P_{\mathrm{fa}}$ for this algorithm.

**Figure 18:**
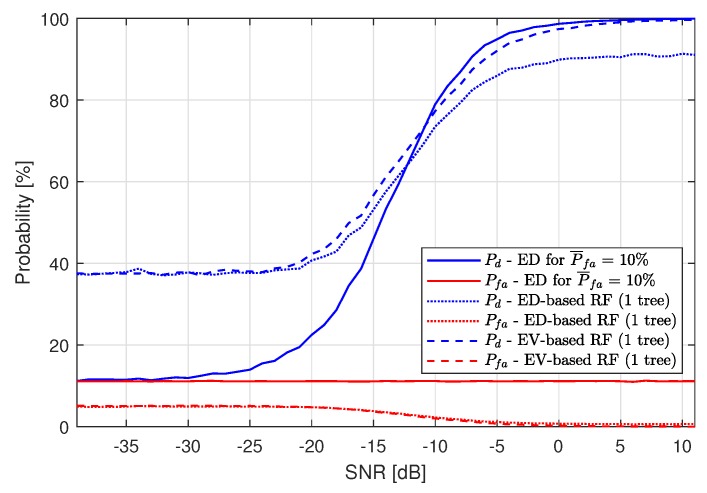
Resulting probabilities $P_{d}$ and $P_{\mathrm{fa}}$ of applied Energy Detection-based Random Forest compared with Energy Vector-based Random Forest for ${\bar{P}}_{\mathrm{fa}}=10\%$ for different SNR values with a shadowing channel.

Finally, Figure 18 presents $P_{d}$ and $P_{\mathrm{fa}}$ for different SNR values in our shadowing scenario considered. In this case, ED improvement can also be observed with the applied RF method, especially for low SNR values.

**Figure 19:**
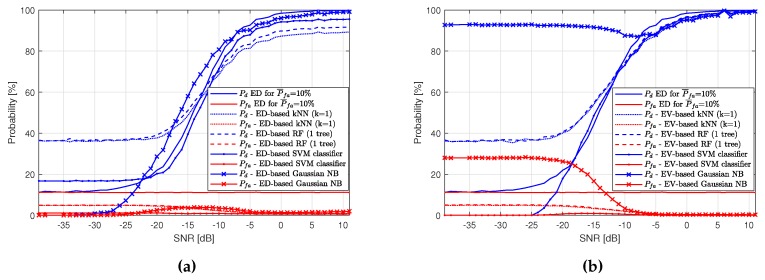
Probabilities $P_{d}$ and $P_{\mathrm{fa}}$ in different locations for the applied k-Nearest Neighbors, Random Forest, Gaussian Naive Bayes and Support Vector Machine classifier methods. (**a**) $P_{d}$ and $P_{\mathrm{fa}}$ surfaces for Energy Detection-based Machine Learning algorithms; (**b**) $P_{d}$ and $P_{\mathrm{fa}}$ surfaces for Energy Vector-based Machine Learning algorithms.

In order to summarize experiment results, a comparison was made between kNN and RF algorithms and algorithms most commonly found in the literature, namely SVM and NB algorithms. Figure 19a presents results of ED-based algorithms: the kNN, the RF, the SVM classifier (with radial basis function kernel) and the Gaussian NB. Similarly, Figure 19b shows results for the same ML methods but EV-based. It can be observed that SVM and Gaussian NB methods do not improve detection performance very much for low SNR values. The exception is the EV-based Gaussian NB method, which greatly improves $P_{d}$ for low SNR values, but unfortunately also introduces a large increase in $P_{fa}$.

## 6. Conclusions

In this paper, the use of machine learning algorithms has been considered for the purpose of increasing the quality of spectrum sensing of LTE downlink signal transmission. In contrast to previously published papers, the greatest emphasis was placed on trying to learn signal dependencies on various domains. By learning some signal’s properties and traffic intensity correlation in time and frequency, it is possible to increase detection probability of the mentioned transmission. We have shown that the considered machine learning methods (kNN and RF), based on energy vectors as the input data, perform better than those based on energy-detection hard decisions, for high SNR values; however, they require more memory and computational capabilities. In order to suppress the need of SNR estimation for choosing the training data in ML algorithms, and also to examine the impact of sensor location, learning algorithms in different locations of the considered area have been implemented. The obtained results show further improvement in terms of the probability of detection and false alarm.

## Figures and Tables

**Table 1:** Comparison of Energy Detection-based Machine Learning and Energy Vector-based Machine Learning.

Energy Detection	Energy Vectors
Advantages	
Can be used adaptively—for low SNR Machine Learning is also used, for high SNR just Energy Detection results are sufficient.	Can be used for every SNR value—results are always better or close to Energy Detection results.
Requires much less memory.	Does not require knowledge on noise or noise estimation.
Disadvantages	
Chosen Energy Detection threshold has a big impact on detection probability.	High computational complexity—Machine Learning used in all transmission conditions.
Knowledge on noise is needed.	Memorization of real numbers required.

## Abbreviations

The following abbreviations are used in this manuscript:

CR	Cognitive Radio
SU	Secondary User
PU	Primary User
SS	Spectrum Sensing
ML	Machine Learning
RB	Resource Block
ED	Energy Detection
ELM	Extreme Learning Machine
SVM	Support Vector Machine
NB	Naive Bayes
kNN	k-Nearest Neighbors
RF	Random Forest
EV	Energy Vector
DT	Decision Tree
AWGN	Additive White Gaussian Noise
EPA	Extended Pedestrian A model
SNR	Signal-to-Noise Ratio
LTE	Long-Term Evolution
OFDM	Orthogonal Frequency Division Multiplexing

## References

[1] The Ericsson Mobility Report. https://www.ericsson.com/en/mobility-report/reports/june-2019.

[2] Yucek T., Arslan H. (2009). A survey of spectrum sensing algorithms for cognitive radio applications. IEEE Commun. Surv. Tutorials.

[3] Enserink S., Cochran D. A cyclostationary feature detector. Proceedings of the 28th Asilomar Conference on Signals, Systems and Computers.

[4] Kapoor S., Rao S., Singh G. Opportunistic Spectrum Sensing by Employing Matched Filter in Cognitive Radio Network. Proceedings of the 2011 International Conference on Communication Systems and Network Technologies.

[5] Kim K., Xin Y., Rangarajan S. Energy Detection Based Spectrum Sensing for Cognitive Radio: An Experimental Study. Proceedings of the IEEE Global Telecommunications Conference GLOBECOM 2010.

[6] Digham F.F., Alouini M., Simon M.K. (2007). On the Energy Detection of Unknown Signals Over Fading Channels. IEEE Trans. Commun..

[7] Kostylev V.I. Energy detection of a signal with random amplitude. Proceedings of the 2002 IEEE International Conference on Communications.

[8] Annamalai A., Olabiyi O., Alam S., Odejide O., Vaman D. Unified analysis of energy detection of unknown signals over generalized fading channels. Proceedings of the 7th International Wireless Communications and Mobile Computing Conference.

[9] Ribas A.O.P., Dias U.S. On the double threshold energy detection-based spectrum sensing over *κ*-*μ* fading channel. Proceedings of the IEEE Radio and Wireless Symposium (RWS).

[10] Verma P., Singh B. Simulation study of double threshold energy detection method for cognitive radios. Proceedings of the 2nd International Conference on Signal Processing and Integrated Networks (SPIN).

[11] Xie S., Shen L. Double-threshold energy detection of spectrum sensing for cognitive radio under noise uncertainty environment. Proceedings of the 2012 International Conference on Wireless Communications and Signal Processing (WCSP).

[12] Farag H.M., Mohamed E.M. Improved Cognitive Radio energy detection algorithm based upon noise uncertainty estimation. Proceedings of the 31st National Radio Science Conference (NRSC).

[13] Hu X., Xie X.Z., Song T., Lei W. An algorithm for energy detection based on noise variance estimation under noise uncertainty. Proceedings of the IEEE 14th International Conference on Communication Technology.

[14] Sequeira S., Mahajan R.R., Spasojević P. On the noise power estimation in the presence of the signal for energy-based sensing. Proceedings of the 35th IEEE Sarnoff Symposium.

[15] Mariani A., Giorgetti A., Chiani M. (2011). Effects of Noise Power Estimation on Energy Detection for Cognitive Radio Applications. IEEE Trans. Commun..

[16] Subekti A., Pardede H.F., Sustika R., Suyoto Spectrum Sensing for Cognitive Radio using Deep Autoencoder Neural Network and SVM. Proceedings of the 2018 International Conference on Radar, Antenna, Microwave, Electronics, and Telecommunications (ICRAMET).

[17] Agarwal A., Dubey S., Khan M.A., Gangopadhyay R., Debnath S. Learning based primary user activity prediction in cognitive radio networks for efficient dynamic spectrum access. Proceedings of the 2016 International Conference on Signal Processing and Communications (SPCOM).

[18] Jan S.U., Vu V.H., Koo I.S. Performance Analysis of Support Vector Machine-Based Classifier for Spectrum Sensing in Cognitive Radio Networks. Proceedings of the 2018 International Conference on Cyber-Enabled Distributed Computing and Knowledge Discovery (CyberC).

[19] Tavares C.H.A., Abrão T. Bayesian estimators for cooperative spectrum sensing in cognitive radio networks. Proceedings of the 2017 IEEE URUCON.

[20] Ma X., Ning S., Liu X., Kuang H., Hong Y. Cooperative Spectrum Sensing using Extreme Learning Machine for Cognitive Radio Networks with Multiple Primary Users. Proceedings of the IEEE 3rd Advanced Information Technology, Electronic and Automation Control Conference (IAEAC).

[21] Mustafa R., Jaglan R.R., Agrawal S. (2019). Decision-fusion-based reliable CSS scheme in CR networks. IET Commun..

[22] Mikaeil A.M., Guo B., Wang Z. Machine Learning to Data Fusion Approach for Cooperative Spectrum Sensing. Proceedings of the 2014 International Conference on Cyber-Enabled Distributed Computing and Knowledge Discovery.

[23] Zhang K., Li J., Gao F. Machine learning techniques for spectrum sensing when primary user has multiple transmit powers. Proceedings of the 2014 IEEE International Conference on Communication Systems.

[24] Awe O.P., Naqvi S.M., Lambotharan S. Variational Bayesian learning technique for spectrum sensing in cognitive radio networks. Proceedings of the 2014 IEEE Global Conference on Signal and Information Processing (GlobalSIP).

[25] Ghazizadeh E., Nikpour B., Moghadam D.A., Nezamabadi-pour H. A PSO-based weighting method to enhance machine learning techniques for cooperative spectrum sensing in CR networks. Proceedings of the 1st Conference on Swarm Intelligence and Evolutionary Computation (CSIEC).

[26] Shalev-Shwartz S., Ben-David S. (2014). Understanding Machine Learning: From Theory to Algorithms.

[27] Cover T., Hart P. (1967). Nearest neighbor pattern classification. IEEE Trans. Inf. Theory.

[28] Müller A.C., Guido S. (2016). Introduction to Machine Learning with Python: A Guide for Data Scientists.

[29] Ho T.K. (1998). The random subspace method for constructing decision forests. IEEE Trans. Pattern Anal. Mach. Intell..

[30] Song L., Shen J. (2010). Evolved Cellular Network Planning and Optimization for UMTS and LTE.

[31] Pedregosa F., Varoquaux G., Gramfort A., Michel V., Thirion B., Grisel O., Blondel M., Prettenhofer P., Weiss R., Dubourg V. (2011). Scikit-learn: Machine Learning in Python. J. Mach. Learn. Res..

[32] Wasilewska M. (2019). Machine Learning for LTE Energy Detection Performance Improvement (Version 1). Data Set.

